# Efficient tumor transduction and antitumor efficacy in experimental human osteosarcoma using retroviral replicating vectors

**DOI:** 10.1038/s41417-018-0037-y

**Published:** 2018-07-25

**Authors:** Shuji Kubo, Misato Takagi-Kimura, Noriyuki Kasahara

**Affiliations:** 1 Unit of Molecular and Genetic Therapeutics, Institute for Advanced Medical Sciences, Hyogo College of Medicine, Nishinomiya, Japan; 2 Departments of Cell Biology and Pathology, Sylvester Comprehensive Cancer Center, University of Miami, Miami, FL USA

**Keywords:** Gene delivery, Cancer therapy, Gene delivery, Cancer therapy

## Abstract

Retroviral replicating vectors (RRVs) have achieved efficient tumor transduction and enhanced therapeutic benefit in a wide variety of cancer models. Here, we evaluated two different RRVs derived from amphotropic murine leukemia virus (AMLV) and gibbon ape leukemia virus (GALV), which utilize different cellular receptors (PiT-2 and PiT-1, respectively) for viral entry, in human osteosarcoma cells. Quantitative RT-PCR showed that low levels of expression of both receptors were observed in normal and non-malignant cells. However, high PiT-2 (for AMLV) and low PiT-1 (for GALV) expression was observed in most osteosarcoma cell lines. Accordingly, AMLV expressing the green fluorescent protein gene infected and replicated more efficiently than GALV in most osteosarcoma cell lines. Furthermore, RRVs expressing the cytosine deaminase prodrug activator gene showed differential cytotoxicity that correlated with the results of viral spread. AMLV-RRV-mediated prodrug activator gene therapy achieved significant inhibition of subcutaneous MG-63 tumor growth over GALV in nude mice. These data indicate that AMLV vectors predominate over GALV in human osteosarcoma cells. Moreover, our findings support the potential utility of the two RRVs in personalized cancer virotherapy on the basis of receptor expression.

## Introduction

Osteosarcoma is the most common primary bone tumor, primarily affecting young children and adolescents [1–5]. It is an aggressive malignant tumor that typically metastasizes to the lungs at an early phase [3–5]. Despite reported increases in survival rates because of improvements in chemotherapy, patients with pulmonary metastases at the time of diagnosis have only a 20% survival rate [2, 3, 5]. Therefore, novel therapeutic paradigms are urgently required.

The use of oncolytic viruses capable of tumor-selective replication has shown considerable promise as a novel treatment strategy [6, 7]. This is based on the observation that tumor cells have impaired antiviral responses making them more sensitive to replicating viruses [8]. However, experimental gene therapy for osteosarcoma has utilized only a few oncolytic viruses in the development of replicating viruses, including adenovirus [9–13], Semliki Forest virus [14], and Maraba virus [15].

Recently, we demonstrated that retroviral replicating vectors (RRVs) show tumor selectivity because of their inherent and stringent specificity for mitotically active cells. In contrast to other viruses used in cancer virotherapy, RRVs are non-cytolytic, but can be engineered to carry prodrug activator genes, which mediate synchronized cell killing of infected tumor cells upon prodrug administration. Using RRVs expressing the yeast cytosine deaminase (CD) prodrug activator gene, we showed highly efficient killing of a wide variety of cancer cells both in vitro and in vivo upon administration of its prodrug, 5-fluorocytosine (5FC) [16–22]. Based on these promising preclinical results, a Phase 1 clinical trial for RRV vector-mediated prodrug activator gene therapy in patients with recurrent high grade glioma was recently conducted in the US and Europe, resulting in complete remission and potential survival benefits [23]. RRV are also currently being evaluated in an international Phase III trial for recurrent high grade glioma (NCT02414165).

To date, we have developed two different RRVs derived from amphotropic murine leukemia virus (AMLV) and gibbon ape leukemia virus (GALV) [22, 24]. Although both AMLV and GALV belong to the gammaretroviruses, they have divergent host ranges and are not in the same interference class [25–27] because they employ different receptors to infect target cells [25, 27]. AMLV uses cellular receptor PiT2 (SLC20A2) while GALV uses PiT1 (SLC20A1); both are mammalian type III inorganic phosphate transporters present in all phyla, which function as ubiquitously expressed facilitators of phosphate uptake [25–27].

In the present study, we compared AMLV-derived and GALV-derived RRVs for prodrug activator gene therapy of experimental human osteosarcoma. Our results indicate the potential utility of AMLV-mediated prodrug activator gene therapy in the treatment of osteosarcoma, including when the tumor is not permissive for GALV.

## Materials and methods

### *PiT-1* and *PiT-2* mRNA expression profile analysis

Total RNA was extracted from semiconfluent cell cultures grown on 10 cm dishes using the Sepasol-RNA I Super G RNA extraction solution (Nacalai Tesque, Inc., Kyoto, Japan). It was then treated with DNase to remove genomic DNA contamination. Quantitative (q)PCR was used to examine the expression of *PiT-1*, *PiT-2*, and glyceraldehyde-3-phosphate dehydrogenase (*GAPDH*) mRNA with the TaqMan One-Step RT-PCR Master Mix Reagents kit (Applied Biosystems Japan Ltd., Tokyo, Japan) according to the manufacturer’s instructions. Primers and TaqMan probes for PiT-1 (Hs00965587_m1), PiT-2 (Hs00198840_m1), and GAPDH (Hs99999905_m1) were purchased from Applied Biosystems Japan. Briefly, 20 ng of total RNA was added to the reaction mixture containing 18 pmol of each of the primers and 5 pmol of probe, and amplified for one cycle of 48 °C for 30 min and 95 °C for 10 min, followed by 50 cycles of 95 °C for 15 s and 60 °C for 1 min.

### Cell lines

Normal human osteoblast cells (NHOsts) and their specific media were purchased from Lonza Japan, Inc. (Tokyo, Japan). Human dermal fibroblasts were purchased from Cell Systems Corporation (Kirkland, WA, USA) and were grown in Roswell Park Memorial Institute 1640 medium (Nacalai Tesque) supplemented with 10% fetal calf serum (FCS; HyClone, Logan, UT, USA). The human embryonic kidney 293 cell line (transformed by the *E1* gene of adenovirus type 5) (HEK293; Microbix, Toronto, Canada) [28] was cultured in Dulbecco’s modified Eagle’s medium (DMEM; Nacalai Tesque) supplemented with 10% FCS. Human osteosarcoma cell lines HOS, MG-63, and Saos-2, were purchased from the RIKEN BioResearch Center (Tsukuba, Ibaraki, Japan), and MNNG-HOS and U2OS human osteosarcoma cell lines were obtained from the American Type Culture Collection (Manassas, VA, USA). These cells were grown in DMEM supplemented with 10% FCS. All cells were incubated at 37 °C/5% CO_2_.

### Viral vector plasmid and virus production

The RRV vector plasmids pAMLV-GFP, pGALV-GFP, pAMLV-CD, and pGALV-CD have been described previously [22]; each contains a full-length replication-competent amphotropic AMLV or GALV provirus with an additional internal ribosome entry site (IRES)-GFP or IRES-CD cassette, respectively (Figs. 2a and 3a). For virus production, 293T cells were transiently transfected with the vector plasmid using Lipofectamin 2000 (Life Technologies Japan, Tokyo, Japan), replenished with serum-free medium, then the supernatant was harvested 48 h later, filtered, and stored at –80 °C [19, 22]. Vector titers were determined by fluorescent protein expression using a FACScalibur flow cytometer (Becton Dickinson Japan, Tokyo, Japan) and expressed as transducing units per mL.

### In vitro replication kinetics of RRVs

To analyze replication kinetics in vitro, virus vector stocks (AMLV-GFP and GALV-GFP) at a multiplicity of infection (MOI) of 0.01 were used to infect various human cell lines at 20% confluency. At serial time points, the cells were trypsinized, one-fourth of the cells were replated, and the remainder were analyzed for GFP expression by flow cytometry as described above.

### In vitro cytotoxicity assay

To quantitatively analyze drug cytotoxicity, triplicate wells containing human MNNG-HOS, HOS, or MG-63 cells (1 × 10^4^ cells/well) pretransduced with AMLV-CD or GALV-CD at an MOI of 0.01 and maintained for 15 days, were cultured in 96-well tissue culture plates with various concentrations of 5FC. On Day 3, viable cell numbers of triplicate cultures were measured by the AlamarBlue method according to the manufacturer’s instructions (Alamar Biosciences, Inc., Sacramento, CA). Briefly, 40 μL of AlamarBlue was aseptically added to the cultures, which were then returned to the incubator for 3 h; fluorescence was measured by an ARVO X4 multilabel plate reader with a 544 nm excitation wavelength and a 590 nm emission wavelength (PerkinElmer Japan, Tokyo, Japan). The percentage of viable cells was determined by calculating the fluorescence of viable cells as measured against wells containing no 5FC.

### Prodrug activator gene therapy in subcutaneous tumor models

BALB/c-nu/nu (nude) mice (Charles River Japan Co., Yokohama, Japan) were bred and maintained under specific pathogen-free conditions. All studies followed protocols approved by the Hyogo College of Medicine Animal Research Committee. Human osteosarcoma xenografts were established in 5-week-old to 6-week-old female nude mice by subcutaneous inoculation of 1 × 10^6^ MG-63 cells into the right dorsal flank. When tumors reached a diameter of ∼5–6 mm, three groups of mice (*n* = 10 per group) were then injected intratumorally with 50 μL of PBS, AMLV-CD, or GALV-CD on Day 0, followed by the intraperitoneal administration of 5FC (500 mg/kg/day) three times a week from Day 14 to Day 34. The mice were observed closely and the tumors were measured twice a week. The tumor volume was calculated as *a* × *b*^2^ × 0.5, where *a* and *b* were the largest and smallest diameters, respectively.

### Statistical analysis

The results are presented as mean ± SD. Statistical significances of differences were calculated using the Student’s *t*-test, and a *P*-value of <0.01 was considered significant in all analyses.

## Results

### mRNA expression of cellular receptors for RRVs

**Fig. 1:**
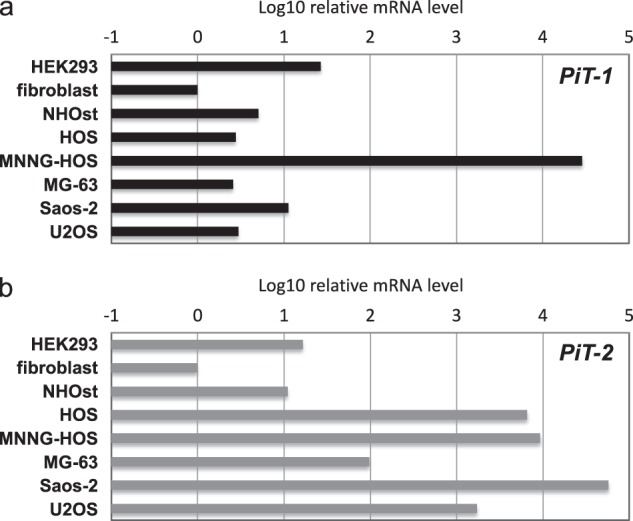
Relative mRNA levels of *PiT-1* and *PiT-2* detected by quantitative RT-PCR. Total RNA was extracted from various human cells, including non-malignant human cells (HEK293 cells, fibroblasts, and NHOsts) and osteosarcoma cell lines (HOS, MNNG-HOS, MG-63, Saos-2, and U2OS). RNA was reverse-transcribed and amplified by PCR with specific primers for PiT-1 (**a**), PiT-2 (**b**), and GAPDH as a control for normalization

We first analyzed the expression levels of cellular receptors for AMLV (PiT-2) and GALV (PiT-1) in human osteosarcoma cell lines by qPCR. In normal human primary NHOsts and fibroblasts and non-malignant human HEK293 cells, mRNA levels of both *PiT-1* and *PiT-2* (Fig. 1a, b) were relatively low, at less than 10.3-fold compared with fibroblasts. In most osteosarcoma cells (HOS, MG-63, Saos-2, and U2OS), low *PiT-1* but high *PiT-2* expression was observed. However, in MNNG-HOS cells, mRNA levels of both *PiT-1* and *PiT-2* were high (>10,000-fold compared with fibroblasts). These findings of low *PiT-1* expression in non-malignant cells but high *PiT-1* expression in most osteosarcoma cells suggested an advantage of AMLV over GALV for targeting human osteosarcoma to ensure safety and efficacy.

### AMLV vectors replicate efficiently in cultured human osteosarcoma cell lines

**Fig. 2:**
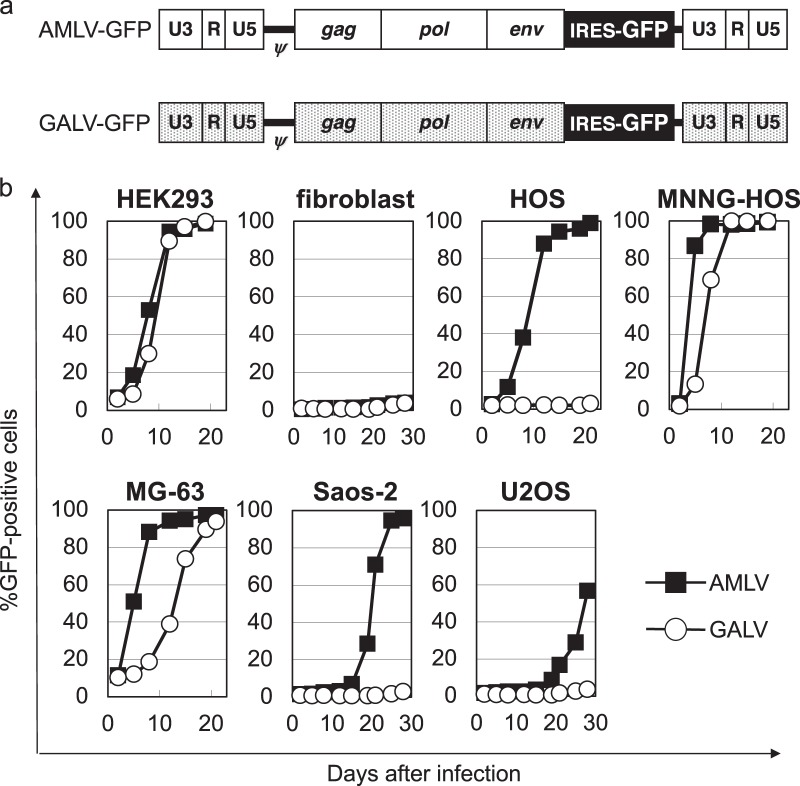
Structure and replication kinetics of amphotropic murine leukemia virus (AMLV) vs. gibbon ape leukemia virus (GALV) vectors in. **a** Schematic structure of AMLV-GFP and GALV-GFP vector. These vectors contain a full-length replication-competent AMLV or GALV provirus, in which an IRES-GFP cassette has been inserted between the *env* gene and 3′-untranslated region. *ψ* packaging signal, *gag-pol* AMLV or GALV structural genes, IRES internal ribosome entry site, GFP green fluorescent protein. **b** Replication kinetics of AMLV vs. GALV vectors in human osteosarcoma cells. Human non-malignant cells (HEK293 cells and fibroblasts) and osteosarcoma cells (HOS, MNNG-HOS, MG-63, Saos-2, and U2OS) were inoculated with AMLV-GFP or GALV-GFP vectors at an MOI of 0.01. On the days of passage, cells were analyzed for GFP expression by flow cytometry. Data are representative of more than three independent experiments, all yielding similar results

We next compared the replication kinetics of AMLV-GFP and GALV-GFP in human osteosarcoma cells (Fig. 2a). Both RRVs efficiently spread up to 90% by 12 days after virus inoculation in non-malignant transformed HEK293 cells (positive control), but did not spread (<5% at 28 days) in normal fibroblasts (negative control), as expected, so we used these cells for controls (Fig. 2b). AMLV-GFP spread more effeciently than GALV-GFP in osteosarcoma cells (HOS, MNNG-HOS, MG-63, Saos-2, and U2OS), although GALV-GFP also spread efficiently in MNNG-HOS cells (Fig. 2b). These results were consistent with those of RRV receptor expression in these cells. Thus, AMLV-RRV predominates over GALV-RRV in human osteosarcoma cells.

### Prodrug activator gene-mediated cell killing effect of RRVs in human osteosarcoma cells

**Fig. 3:**
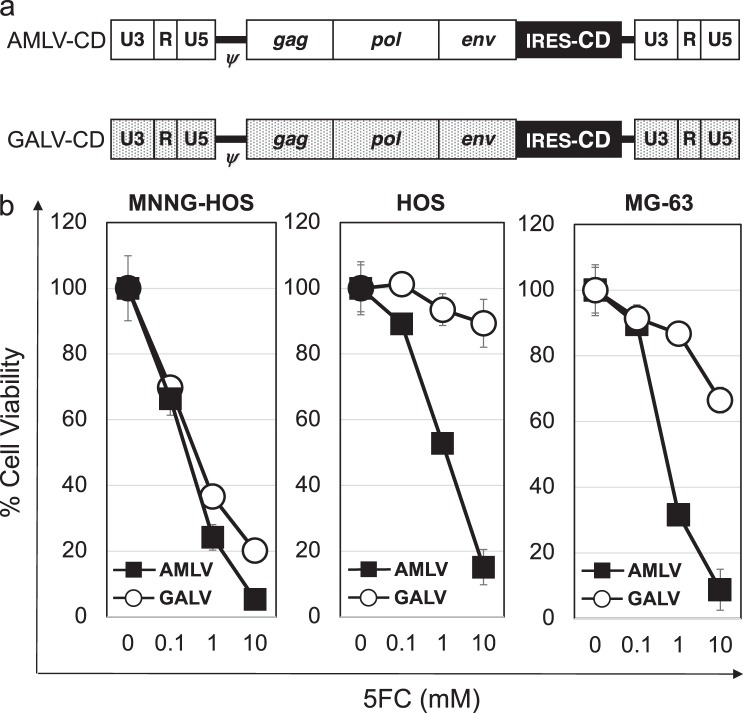
Prodrug activator gene-mediated cell killing effect after AMLV vs. GALV infection in vitro. **a** Schematic structure of AMLV-CD and GALV-CD vector. These vectors were created by replacement of the IRES-GFP cassette of AMLV-GFP and GALV-GFP with IRES-CD, respectively. CD yeast cytosine deaminase prodrug activator gene. **b** Cell viability of RRV-infected osteosarcoma cells. MNNG-HOS, HOS, and MG-63 were infected with GALV-CD or AMLV-CD at an MOI of 0.01 on Day 0, and exposed to various concentrations of the 5FC prodrug from Day 15 for 3 days. On Day 18, cell viability was examined by the AlamarBlue assay. Data shown are averages ± SD from experiments performed in triplicate

To investigate the efficacy of RRV-mediated prodrug activator gene therapy in human osteosarcoma cells, we used AMLV-CD and GALV-CD which express the CD prodrug activator gene (Fig. 3a). The human osteosarcoma cells MNNG-HOS, HOS, and MG-63 were infected with MALV-CD or GALV-CD at an MOI of 0.01 on Day 0, and exposed to various concentrations of the 5FC prodrug from Day 15 for 3 days. On Day 18, cell viability was examined by the AlamarBlue assay. In MNNG-HOS cells (high *PiT-1* and high *PiT-2* expression), decreased cell viability was observed in both AMLV-CD-transduced and GALV-CD-transduced cultures in a 5FC dose-dependent manner (Fig. 3b). However, in HOS and MG-63 cells (low *PiT-1* and high *PiT-2* expression), significantly decreased cell viability was observed in AMLV-CD-transduced compared with GALV-CD-transduced cultures.

These results were consistent with the in vitro RRV-GFP replication kinetics (Fig. 2b), and indicate that the AMLV vector could achieve selective spread and cytotoxicity in osteosarcoma cells, as well as enhanced transduction efficiency by using a different physical binding mechanism.

### AMLV-mediated CD/5FC prodrug activator gene therapy shows potent in vivo antitumor effects in a subcutaneous human osteosarcoma xenograft model

**Fig. 4:**
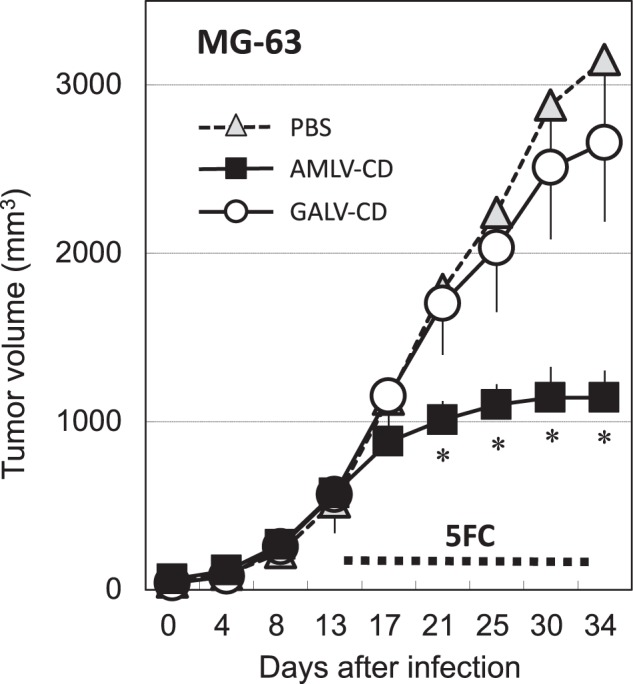
In vivo antitumor effect of AMLV vs. GALV-mediated prodrug activator gene therapy in a subcutaneous xenograft model of human osteosarcoma cells. MG-63 tumors were grown subcutaneously in nude mice to 5–6 mm in diameter, then injected intratumorally with 1 × 10^4^ TU (50 μL) of either AMLV-CD or GALV-CD, or PBS vehicle control on Day 0, followed by intraperitoneal administration of 5FC (500 mg/kg/day) three times weekly from Day 14 to Day 34 (*n* = 10 per group). Tumor volumes were measured twice weekly, and data shown are averages—(PBS and GALV-CD) or +(AMLV-CD) SD from experiments. *P < 0.01 (AMLV-CD vs. GALV-CD)

To examine the antitumoral therapeutic efficacy of RRV-mediated CD/5FC prodrug activator gene therapy, nude mice bearing established MG-63 tumors were treated with a single intratumoral injection (1 × 10^4^ TU total dose) of either AMLV-CD or GALV-CD, or PBS vehicle control on Day 0, followed by intraperitoneal administration of 5FC. As shown in Fig. 4, subcutaneous tumors treated with GALV-CD showed no obvious inhibition of growth after 5FC administration compared with the PBS control group. In contrast, the growth of AMLV-CD-transduced tumors was significantly inhibited by 5FC treatment (*P* < 0.01 after Day 21). Thus, AMLV-mediated prodrug activator gene therapy achieved effective in vivo growth inhibition in this subcutaneous MG-63 tumor model of human osteosarcoma.

## Discussion

To achieve successful prodrug activator gene therapy for cancer, efficient transduction throughout the entire tumor is required. Replicative spread of RRVs in solid tumors are potentially affected by many factors, including cell proliferation rate, antiviral innate immunity, acquired immunity, and cellular receptor expression levels. Among the factors, we previously reported that the replicative spread of RRVs in solid tumors is affected by cellular receptor expression levels in human malignant mesothelioma cells [22]. In the previous study, we detected high *PiT-1* expression compared with *PiT-2*, suggesting that GALV predominates over AMLV in these cells, especially in ACC-MESO-1 cells [22]. In this study, however, we observed low *PiT-1* expression compared with *PiT-2*, indicating that AMLV predominates over GALV in human osteosarcoma cells. These results demonstrate that some cancer cell lines express PiT-1 and PiT-2 differentially, although the mechanisms and physiological effects of differential expression of phosphate transporters remain to be elucidated. In cells with low receptor expression, RRVs that require this receptor for viral entry showed limited replicative spread, and the efficiency of cell death after 5FC administration was correlated with RRV replication kinetics. Together with the evaluation for other factors in biopsy specimens, the use of multiple RRVs may be practically beneficial in RRV-mediated prodrug activator gene therapy against a variety of solid tumors. Moreover, personalized virotherapy based on cellular receptor expression could enhance efficacy when targeting human solid tumors.

Although most osteosarcomas have a good response to chemotherapy, ∼30–40% of the patients eventually develop metastases. Moreover, >80% of relapses involve the lungs [1], and patients with pulmonary metastases at the time of diagnosis have only a 20% survival rate [2, 3, 5]. RRV has been shown to effectively transduce multiple disseminated tumors [19, 29] and achieve strong therapeutic effects in mouse models, probably due to a potent bystander effect that we previously reported using the same vector as we used in this study [16, 17]. Hiraoka et al. reported variable transduction levels among different tumor nodules after the intrasplenic injection of RRV vector in hepatic metastasis models of murine CT26 colorectal cancer [29]. Kawasaki et al. documented the efficient transduction of human malignant mesothelioma cells by RRV vector and therapeutic efficacy of RRV-mediated CD/5FC prodrug activator gene therapy in peritoneally disseminated mesothelioma models using human MSTO-211H cells pretransduced with AMLV-CD [19].

In multiple (disseminated or metastatic) tumors, the development of alternative methods for efficient in vivo systemic delivery of RRV to tumor sites is desirable, because retrovirus-based vectors are produced at only low titers in vitro, are hugely diluted in the systemic circulation, and are easily inactivated by complement in the blood. To this end, we developed a novel chimeric vector system (adenovirus–RRV hybrid vectors), in which high-titer adenoviruses were used to deliver RRV vectors. The hybrid vector system exhibited a significantly higher initial transduction and higher levels of second-stage RRV production in situ, leading to accelerated RRV vector spread and achieving enhanced therapeutic efficacy of prodrug activator gene therapy for cancer [30]. This hybrid vector may also have additional advantages for tumor targeting via fiber modification of the first-stage adenovirus [31, 32] and transcriptional regulation of second-stage RRV vectors [33]. This system therefore has great therapeutic potential to improve the clinical outcome in patients with aggressive osteosarcomas.

In conclusion, our results show that RRV vectors can efficiently replicate and achieve significant levels of tumor transduction in human osteosarcoma cells. This is the first study to show efficient transduction of human osteosarcoma cells by RRV vectors, as well as the therapeutic efficacy of AMLV vector-mediated CD/5FC prodrug activator gene therapy in an MG-63 osteosarcoma model not permissive to GALV. As such, this system could be a new treatment paradigm for human osteosarcoma.
